# Second-line therapy in testicular germ cell tumours: results from a tertiary cancer care centre in India

**DOI:** 10.3332/ecancer.2022.1408

**Published:** 2022-06-13

**Authors:** Amit Joshi, Devanshi Kalra, Vijai Simha, Nandini Menon, Vanita Noronha, Ganesh Bakshi, Gagan Prakash, Mahendra Pal, Vedang Murthy, Santosh Menon, Nilesh Sable, Archi Agrawal, Pallavi Rane, Kumar Prabhash

**Affiliations:** 1 Department of Medical Oncology, Tata Memorial Hospital, Homi Bhabha National Institute (HBNI), Mumbai 40012, India; 2 Department of Surgical Oncology, Tata Memorial Hospital, Homi Bhabha National Institute (HBNI), Mumbai 40012, India; 3 Department of Radiation Oncology, Tata Memorial Hospital, Homi Bhabha National Institute (HBNI), Mumbai 40012, India; 4 Department of Pathology, Tata Memorial Hospital, Homi Bhabha National Institute (HBNI), Mumbai 40012, India; 5 Department of Radiology, Tata Memorial Hospital, Homi Bhabha National Institute (HBNI), Mumbai 40012, India; 6 Department of Nuclear Medicine, Tata Memorial Hospital, Homi Bhabha National Institute (HBNI), Mumbai 40012, India

**Keywords:** testicular cancer, second line therapy, TIP

## Abstract

**Background:**

Malignant testicular neoplasms constitute about 1% of all cancers in males. This is one of the most common tumours in adolescents and young adult males. After the introduction of cisplatin-based chemotherapy, the survival of germ cell tumour patients, even those with poor prognostic risk factors, has significantly improved over the years. Second-line chemotherapy in patients who have progressed over the first-line cisplatin-based chemotherapy has shown convincing 5 years of overall survival (OS).

**Methodology:**

This study is a retrospective analysis of testicular cancer patients from 2014 to 2020 who have received salvage chemotherapy treatment at Tata Memorial Centre. Patient demographics, tumour characteristics and treatment details were recorded in a specific format, and progression-free survival and OS were analysed along with response to therapy.

**Results:**

A total of 46 testicular cancer patients from 2014 to 2020, who received second-line chemotherapy, were analysed from the database maintained at our hospital. The median age at diagnosis was 29.5 (18–60) years. Most of the patients (30, 65.2%) presented with lung metastasis and 11 (23.9%) patients with liver metastasis. Most of the patients (21, 45.6%) received vinblastine, ifosfamide and cisplatin, whereas 13 (28.2%) patients received paclitaxel, ifosfamide and cisplatin regimen and 7 (15.2%) patients received GemOx regimen as the second-line chemotherapy. Median OS was observed to be 33.97 months and median progression-free survival was 29.01 months.

**Conclusion:**

Second-line chemotherapy in testicular germ cell tumours can result in long-term disease control and all patients who are fit to tolerate second-line therapy should be offered it. Patients with relapsed seminoma did better than relapsed non-seminomatous germ cell tumours.

## Background

Testicular cancer is one of the most common tumours in adolescents and young adult males. Malignant testicular neoplasms constitute about 1% of all cancers in males [1]. They are classified according to the histological types of the cells, seminomatous and non-seminomatous [2]. Testicular cancers are highly chemotherapy-sensitive tumours. Especially after introducing cisplatin-based chemotherapies, the disease can be cured in 50%–60% of the cases, even if it presents as an advanced disease with poor prognostic risk factors [2].

Of all the testicular cancers, 95% are germ cell tumours [1]. Among these, about 50% are non-seminomatous germ cell tumours (NSGCT). It is one of the most common cancers in young men. The age-standardised incidence rate of testicular cancer is 6.7 and 5.6 per 100,000 population for Europe and the United States, respectively, whereas it is 0.5 per 100,000 population in India [2]. Globally, India has the lowest incidence, and hence there is a lack of literature regarding Indian data on testicular germ cell tumours.

When it comes to the management of relapsed testicular cancer patients, various options are available, such as conventional-dose chemotherapy, i.e., paclitaxel + ifosfamide + cisplatin (TIP) and etoposide + ifosfamide + cisplatin (VIP), and high-dose chemotherapy (HD-CT) regimens, such as carboplatin + etoposide and paclitaxel + ifosfamide + carboplatin + etoposide, followed by autologous bone marrow transplant [3].

Most patients receive second-line salvage chemotherapy after the progression of the disease over first-line therapy; an autologous bone marrow transplant is not feasible because of the unavailability of expertise, cost constraints and also due to lack of any randomised trial showing the superiority of one approach to another one [3].

In this article, we report a single-centre experience of managing testicular cancer patients who have received salvage second-line chemotherapy after the progression of the disease. We, at this moment, would like to describe the clinical presentation, diagnosis, treatment complexities and disease-free survival in the context of the patients who received second-line chemotherapy.

## Materials and methods

### Selection of cases

Case records of testicular cancer patients from 2014 to 2020 were retrospectively reviewed. Patients who had completed second-line chemotherapy were identified. Various parameters, such as patient demographics, tumour characteristics, treatment details recorded in a specific format, progression-free survival and overall survival (OS), were analysed.

### Statistical analysis

Statistical analysis was carried out using Statistical Package for the Social Sciences (SPSS) version 25 (IBM SPSS Statistics, Armonk, NY: IBM Corp). A descriptive analysis was carried out to analyse the demographic characteristics. Kaplan–Meier’s survival analysis computed the progression-free survival and OS.

## Results

Forty-six testicular cancer patients from 2014 to 2020 who have received second-line chemotherapy were analysed from the database maintained at our hospital. All the patients were included for demographic analysis. The patients who had completed at least 1 year of follow-up after second-line treatment were analysed for progression-free and OS. The demographic characteristics were analysed descriptively using SPSS version 25.

### Demographics

The median age was 29.5 years (18–60 years). 40 (87%) patients were diagnosed with NSGCT, whereas 6 (13%) patients were diagnosed with seminomatous germ cell tumours (Table 1).

21 (45.7%) patients had poor risk of the disease, 15 (32.6%) patients had intermediate risk and 10 (21.7%) patients had reasonable risk at the time of initial presentation. 12 (30%) non-seminomatous testicular cancer patients showed partial response (PR) to first-line chemotherapy and 2 (33.%) seminomatous testicular cancer patients had a partial answer to the first-line chemotherapy.

The median time to relapse after completion of first-line chemotherapy is 5.3 months (4.3–90.1 months).

### Metastasis

Most of the patients (30, 65.2%) presented with lung metastasis, 11 (23.9%) patients with liver metastasis and 35 (76%) patients with nodal metastasis with the size of >5 cm in 8 patients and 13 (28.2%) patients had a nodal size between 2 and 5 cm.

Moreover, two patients had brain metastasis before starting second-line chemotherapy, and four developed it after or during second-line chemotherapy.

### Second-line chemotherapy details

Most of the patients (20, 43.4%) received vinblastine, ifosfamide and cisplatin (VeIP) regimen as per the physician’s decision, whereas 16 (34.7%) patients received TIP (paclitaxel, ifosfamide, cisplatin) regimen and 7 (15.2%) patients received GemOX (gemcitabine, oxalipaltin) regimen as the second-line chemotherapy. One of the patients underwent a transplant.

Most of the patients, 15 out of 20 (75%), completed 4 cycles of VIP, whereas 9 out of 16 (56%) patients completed 4 cycles of TIP as second-line chemotherapy at our centre. Progressive disease was the most common cause of not completing the planned therapy.

## Toxicities

Second-line chemotherapy was relatively well tolerated. However, various toxicities are seen during treatment with second-line chemotherapy. Different toxicities were reported, i.e., 7 (15.2%) and 2 (0.043%) patients had developed grade III and grade IV anaemia, and 5 (10.8%) and 10 (21.7%) patients had developed grade III and grade IV neutropenia, respectively. Also, 9 (19.6%) and 3 (6.5%) patients had developed grade III and grade IV thrombocytopenia, respectively. Moreover, grade III diarrhoea and grade III hyponatraemia were reported in 2 (4.3%) patients.

10 (21.7%) patients underwent retroperitoneal lymph node dissection (RPLND) after completing second-line chemotherapy. Based on histopathology, three patients had mature teratoma and three had the viable disease. Also, four patients had no viable disease.

After completion of the second line of chemotherapy, currently, 18 patients are alive (39.1%), out of which 4 (22.2%) are active with the disease and 14 (77.7%) are alive without disease.

Patients with seminomatous testicular cancer showed better response to second-line chemotherapy than patients with non-seminomatous testicular cancer.

We tried to assess the correlation of survival with various prognostic factors. The univariate correlation was observed with elevated tumour markers, such as beta-hCG, alpha-fetoprotein (AFP) and lactate dehydrogenase (LDH), at the time of relapse on first-line therapy, i.e., only beta-hCG levels more than 1,000 at the time of relapse was found to be associated with worse survival (Table 2).

### Response to the first line of chemotherapy

Out of 46 patients, 10 (21.7%) responded thoroughly to the first-line chemotherapy. 9 (22.5%) patients with NSGCT had a complete response (CR), whereas 1 (16.6%) patient with seminoma had a CR after first-line chemotherapy. PR was seen in 12 (30%) NSGCT patients and 2 (33.3%) seminoma patients. 22 (47.8%) patients had progressive disease after the first line of chemotherapy.

### Response to the second line of chemotherapy

Of a total of 46 patients, 7 (15.2%) patients had a stable disease, 5 (10.8%) patients showed CR and 18 (39.1%) patients had a partial answer to the second-line chemotherapy. Also, 10 (21.7%) patients progressed on second-line chemotherapy.

### Survival and disease-free interval

**Figure 1.**
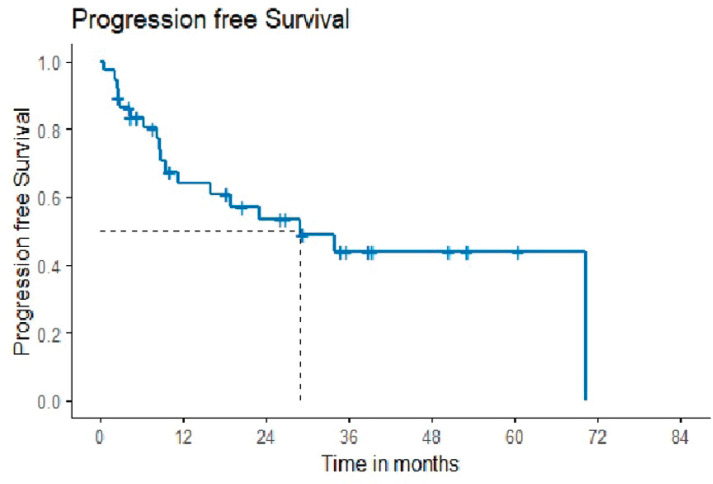
Progression-free survival.

10 (21.7%) out of 46 patients progressed after the second line of therapy. The patients who were lost to follow-up before completing 1-year post-second-line treatment, i.e., 9 out of 46 patients, were not included in the survival analysis. Median progression-free survival was 29.01 months, with a 2-year probability of survival of 53.4 months (35.7–71.1) (Figure 1).

**Figure 2.**
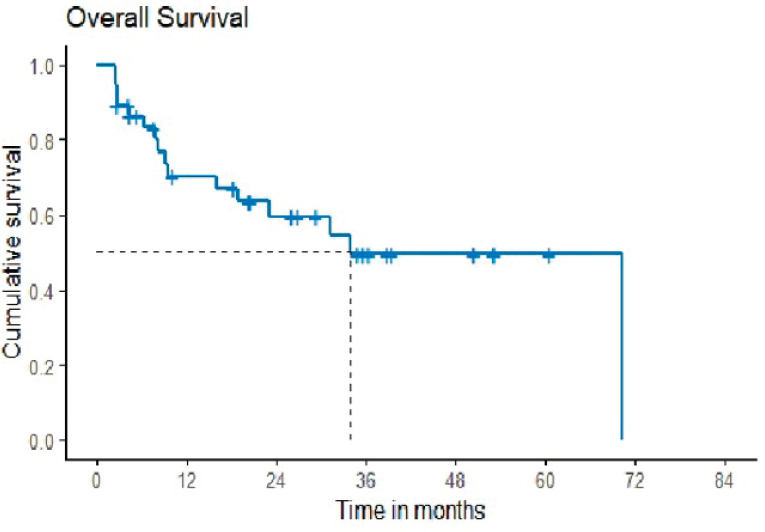
Overall survival.

OS was defined as the time from the start of second-line chemotherapy to the last follow-up date or date of death. The median OS was 33.97 months with 2 years probability of 59.6 months (42.2–76.9) (Figure 2). It was reported that the study’s median follow-up was 34.8 months.

## Discussion

Despite the low incidence of testicular cancer in India and all the advances made in treatment options for testicular cancer, some patients had a recurrence of the disease. There is a lack of literature regarding the outcome of testicular cancer, especially with the role of second-line chemotherapy in treating this type of rare cancer. Hence, to understand the role of second-line chemotherapy and various factors affecting the treatment of patients undergoing second-line chemotherapy in our patient population, we conducted this retrospective analysis of second-line chemotherapy among testicular patients at a tertiary care centre in a developing country.

Our study specified 46 patients given second-line chemotherapy; out of this, 9 patients were lost to follow-up within 1 year of receiving therapy and were not included in the survival analysis. The study by Pico *et al* [4], included 263 patients randomised into 2 arms, and the survey by Loehrer *et al* [5] was conducted on 135 patients. Also, a study by Farhat *et al* [6] included 54 patients, signifying complete remission in 24 patients at completion of VIP chemotherapy (as mentioned in Table 3).

Using one of the predictive models to risk stratify our patients, i.e., Beyer *et al*’s [14] scoring method, 22 (47.8%) patients had good risk, 23 (50%) had intermediate risk and 1 (2.1%) had poor risk. Out of these, CR or PR to second-line chemotherapy was observed in 11 (50%) patients with good risk, 12 (52.1%) with intermediate risk, which is comparable to the study by Beyer *et al* [7] specifying 76.5% of the patients with good risk showing CR/PR and 58.6% of the patients with intermediate risk showing PR/CR to second-line chemotherapy.

Kondagunta *et al* [8] stated a median OS of 42 months. A study by McCaffrey *et al* [7] recorded a median OS of 18 months.

A few initial reports from the American and European groups that included VIP therapy in the second line demonstrated a CR in 25%–36% of the cohort. McCaffrey *et al*’s [7] study included 56 patients, and 36% of the patients had a CR. On the contrary, our study included only 10.8% of the patients who showed CR when given second-line chemotherapy. This difference in response to dual-line salvage therapy may be because, in our setting, we have analysed data of all the patients who have received salvage therapy irrespective of the timing of relapse (platin sensitive versus refractory disease), site of relapse (both visceral and non-visceral), different salvage chemotherapy protocol used (TIP/VI/GemOx) and performance status (PS) of patients [7].

Our study stated that 14 out of 46 patients had a late relapse. Late relapse refers to patients who relapsed after more than 2 years after completion of treatment [9]. Moreover, there is a lack of literature highlighting the late relapses in testicular cancer. Most relapses occur within the first 2 years of therapy; late relapse beyond 2 years is rare [9, 10].

Also, our study concludes that a total of 18 patients out of 46 are currently alive after completion of second-line chemotherapy, out of which 4 patients are active with the disease and the remaining 14 patients are alive without the disease; this is even after 9 patients were lost to follow-up within 1 year. A study by McCaffrey *et al* [7] specified that 23 patients out of 56 were alive; 19 of them were alive without any disease and 4 were active with the disease after completion of second-line chemotherapy.

Previous studies have shown moderately promising results in utilising HD-CT in the salvage treatment of refractory or relapsing germ cell tumours. Still, high-level evidence supporting HD-CT as a second or further-line treatment is lacking. One of the retrospective studies by Lorch *et al* [11] reported a favourable response rate of 55% after HD-CT as a second salvage treatment, with a remarkably long-term survival rate of 17%.

Although the standard second-line salvage therapy is still debatable, peripheral blood stem cell transplantation is also a salvage therapy option. It is commonly used in some parts of the world, especially in medical oncology centres in the USA.

The TIGER trial (A031102, E1407) is an ongoing trial with international collaboration of many centres in North America, Europe and Australia to determine the optimal initial salvage chemotherapy approach in patients with advanced germ cell tumour, which will help in giving a conclusion that either HD-CT, followed by autologous transplant, or salvage chemotherapy alone can be the standard of care in the initial salvage setting [12, 13].

The limitation of our study was that it is a small retrospective study; it does not include various factors that would be better recorded in a prospective study.

Most testicular cancer patients presented with advanced stages. Even with standard BEP (bleomycin, etoposide cisplatin) therapy and getting cured, only a few patients relapse, which can be successfully salvaged in a quarter of the patients and may get long-term control with acceptable toxicity at a community practice level.

## Conclusion

Our study reports the various demographic factors of testicular patients who received second-line chemotherapy in the Indian context. Second-line chemotherapy in testicular germ cell tumours can result in long-term disease control. The high durable response proportion emphasises the importance of patient selection according to prognostic factors such as histopathology and tumour markers. It should be offered to all patients who have progressive disease on first-line therapy with good PS.

## Conflicts of interest

None.

## Funding

None.

## Disclosure of results at a meeting

None.

## Figures and Tables

**Table 1.** Demographic characteristics.

Characteristics	Value-number (percentage)
**Age (years)**	
Range	18–60
Median	29.5
**Baseline diagnosis**	
NSGCT	40 (87%)
Seminoma	6 (13%)
**Risk (at time of diagnosis)**	
Good risk	10 (21.7%)
Intermediate risk	15 (32.6%)
Poor risk	21 (45.7%)
**Metastasis at diagnosis**	
Lung metastasis	30 (65.2%)
Liver metastasis	11 (23.9%)
Nodal metastasis	35 (76%)
**Tumour markers at end of first-line chemotherapy**	
AFP (>100)	11 (23.9%)
Beta-hCG (>1,000)	3 (6.5%)
LDH (>2 ULN)	5 (10.8%)

**Table 2.** Prognostic factors and OS.

Prognostic factors	Univariate (*p*-value)
1. AFP ≤ 100	0.665
AFP ≥ 100	
2. Beta-hCG ≤ 1,000	0.001
Beta-hCG ≥ 1,000	
3. LDH ≤ 2 times ULN	0.45
LDH ≥ 2 times ULN	
4. Risk of disease	0.617

**Table 3.** Studies conducted in refractory testicular cancer patients.

Authors	Number of patients	Chemotherapy regimen	OS/PFS
Pico *et al* [4]	263	Arm A (4PEI/VeIP) arm B (3PEI/VeIP followed by CarboPEC)	PFS = 3 years
Loehrer *et al* [5]	135	VeIP	Median PFS = 4.7 years
Kondagunta *et al* [8]	48	Paclitaxel/ifosfamide	Median OS = 42 months
McCaffrey *et al* [7]	56	VeIp/VIP	Median OS = 18 months
Farhat *et al* [6]	54	VIP/VeIP	Median PFS = 6 months
Joshi *et al* (our study)	46	VeIP/TIP/GemOx	Median OS = 33.97 months, Median PFS = 29.01 months

OS: overall survival; PFS: progression-free survival
